# The molecular biology of tubulinopathies: Understanding the impact of variants on tubulin structure and microtubule regulation

**DOI:** 10.3389/fncel.2022.1023267

**Published:** 2022-11-02

**Authors:** Katelyn J. Hoff, Andrew J. Neumann, Jeffrey K. Moore

**Affiliations:** Department of Cell and Developmental Biology, University of Colorado Anschutz Medical Campus, Aurora, CO, United States

**Keywords:** tubulinopathy, microtubule, neurodevelopment, cytoskeleton, protein structure

## Abstract

Heterozygous, missense mutations in both α- and β-tubulin genes have been linked to an array of neurodevelopment disorders, commonly referred to as “tubulinopathies.” To date, tubulinopathy mutations have been identified in three β-tubulin isotypes and one α-tubulin isotype. These mutations occur throughout the different genetic domains and protein structures of these tubulin isotypes, and the field is working to address how this molecular-level diversity results in different cellular and tissue-level pathologies. Studies from many groups have focused on elucidating the consequences of individual mutations; however, the field lacks comprehensive models for the molecular etiology of different types of tubulinopathies, presenting a major gap in diagnosis and treatment. This review highlights recent advances in understanding tubulin structural dynamics, the roles microtubule-associated proteins (MAPs) play in microtubule regulation, and how these are inextricably linked. We emphasize the value of investigating interactions between tubulin structures, microtubules, and MAPs to understand and predict the impact of tubulinopathy mutations at the cell and tissue levels. Microtubule regulation is multifaceted and provides a complex set of controls for generating a functional cytoskeleton at the right place and right time during neurodevelopment. Understanding how tubulinopathy mutations disrupt distinct subsets of those controls, and how that ultimately disrupts neurodevelopment, will be important for establishing mechanistic themes among tubulinopathies that may lead to insights in other neurodevelopment disorders and normal neurodevelopment.

## Introduction

Microtubules are a critical component of every eukaryotic cell, and they have highly regulated roles in multiple neuronal functions during neurodevelopment. During neurodevelopment, microtubules play a key role in the neurons that structure the sulci and gyri of the cerebral cortex. Mutations in the genes encoding the α- and β-tubulin subunits of microtubules are associated with a range of malformations of the cerebral cortex and constitute a class of disorders known as “tubulinopathies.” Cortical malformations encompass multiple types of abnormalities, including too many or too few brain folds, with or without a reduction in brain size. While the number of tubulinopathy mutants identified in patients continues to increase, the molecular mechanisms that connect tubulin mutations to neurodevelopment disorders remains an important area of investigation.

The goal of this review is to discuss the importance of understanding basic tubulin biology and regulatory mechanisms to better predict the neurodevelopmental impacts of individual tubulin mutants. We will discuss foundational literature as well as recent advances in understanding the interconnected role of tubulin structure, microtubules, and microtubule-associated proteins (MAPs) in neurodevelopment. Additionally, we will review the roles of MAPs and motors in both healthy and disease models to understand how microtubules are regulated during neurodevelopment, and how tubulinopathy mutations disrupt this important regulation. Finally, we attempt to identify mechanistic themes between types and locations of tubulinopathy mutations within the protein and the associated cortical malformation.

## Tubulin biology

### Microtubule basics

The cytoskeleton is comprised of various filamentous structures, including microtubules, F-actin, and intermediate filaments. Each of these structures plays critical roles in development, disease, and basic cell biology. Microtubules are polar, cylindrical structures that are highly dynamic and are composed of tubulin heterodimers, consisting of one α and one β monomer. Microtubules are the only cytoskeletal filaments that exhibit what is known as “dynamic instability,” the stochastic cycling between states of polymerization and depolymerization. This dynamic instability is an intrinsic feature of microtubules and can be reconstituted *in vitro* using purified tubulin proteins, independent of any accessory proteins (Mitchison and Kirschner, 1984). Under these conditions, the rate of microtubule polymerization depends on the concentration of free tubulin available in the system, while the rate of depolymerization is concentration-independent. These intrinsic properties of microtubule dynamics are controlled by tubulin’s ability to act as a switch that alternates between conformational states that either favor or disfavor microtubule assembly. In cells, this switch-like behavior of tubulin is further modulated by a host of MAPs and motors.

**FIGURE 1:**
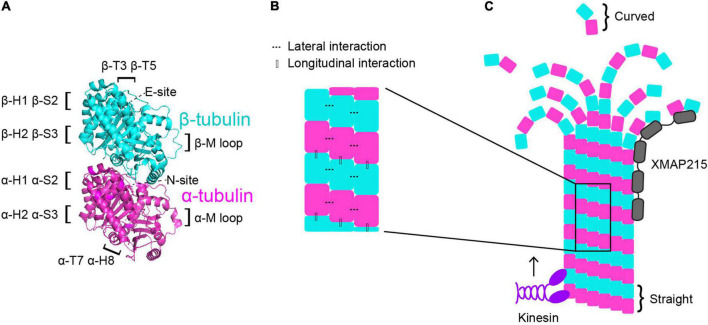
Microtubule dynamics are regulated at multiple control points. **(A)** α-tubulin is human TUBA1A in magenta and β-tubulin is human TUBB3 is in cyan (PDB structure: 5JCO). Regions that participate in longitudinal and lateral interactions are described along the sides of the structure. α- and β-tubulin each bind one GTP molecule. The non-exchangeable “N-site” on α-tubulin sits at the intradimer surface and the GTP is not hydrolyzed. The exchangeable “E-site” on β-tubulin is at the interdimer interface and the GTP molecule is hydrolyzed upon incorporation of the heterodimer into the microtubule lattice. **(B)** Lateral interactions occur between neighboring protofilaments and are illustrated by the dotted lines. Longitudinal interactions occur between at the interdimer interface between two stacked heterodimers. **(C)** Microtubule-associated proteins (MAPs) and motors can have specific preferences for associating with different segments of microtubules. For example, XMAP215 (gray) associates at microtubule plus ends and kinesins (purple) walk along the lattice toward the plus end. Tubulin undergoes a series of conformational changes. Free, curved heterodimer subsequently straightens as it is assembled into microtubule lattice.

One way in which the tubulin heterodimer acts as a switch is via its nucleotide state. Tubulin binds to two GTP molecules at two distinct locations (Figure 1A). One of these sites is the non-exchangeable site, also known as the “N-site,” which is located at the intradimer interface between the α- and β-tubulin monomers (Nogales et al., 1998). As the name suggests, this GTP molecule is not hydrolyzed (Spiegelman et al., 1977). The other GTP-binding site is the exchangeable site, or “E-site.” The E-site is located on β-tubulin where it interacts with the α-tubulin of the incoming tubulin heterodimer during microtubule assembly and can be hydrolyzed from GTP to GDP (Carlier and Pantaloni, 1981; Nogales et al., 1998). Therefore, when referring to the nucleotide state of a heterodimer, it is in reference to the nucleotide state at the E-site, as that is the one that cycles between GTP and GDP and has consequences for microtubule dynamics. GTP-tubulin in solution is added on to the microtubule plus end and exhibits faster assembly activity than GDP-tubulin. As the GTP-tubulin heterodimer is further incorporated into the microtubule, GTP is hydrolyzed to GDP (Nogales et al., 1998; Carlier and Pantaloni, 1981). This process of hydrolysis is coupled to changes in tubulin structure and interaction with its neighbors, and it is primarily thought that GTP-tubulin stabilizes microtubules while GDP-tubulin is less stable (Alushin et al., 2014; Zhang et al., 2015). As GTP-tubulin assembles onto the microtubule plus end, it forms what is known as the “GTP-cap”, which is a stable structure that prevents microtubule depolymerization until the cap is hydrolyzed to GDP (Mitchison and Kirschner, 1984; O’Brien et al., 1987; Drechsel and Kirschner, 1994; Caplow and Shanks, 1996; Roostalu et al., 2020). The formation of the GTP-cap requires that the rate of heterodimer(s) addition at the plus-end is faster than the rate of GTP hydrolysis. Once the rate of GTP hydrolysis outpaces the addition of new heterodimer, the stable GTP-cap is extinguished and the exposed, unstable GDP heterodimers trigger microtubule catastrophe. Additionally, there are conformational changes undergone by the tubulin heterodimer as it undergoes GTP hydrolysis and is incorporated further into the microtubule lattice. Many studies have sought to understand whether tubulin compaction occurs within the microtubule lattice and whether compaction is influenced by nucleotide state and vice versa. For a more comprehensive analysis on tubulin compaction in the microtubule lattice, we direct readers toward previous works (Zhang et al., 2015; Estévez-Gallego et al., 2020; Cleary and Hancock, 2021).

A second way that the tubulin heterodimer acts like a switch to modulate microtubule dynamics involves nucleotide-independent conformational transitions (Figure 1C). Free tubulin heterodimers are curved in solution, while dimers within the microtubule lattice are straight. Early work postulated that the curved vs straight conformations of tubulin were dependent on the nucleotide state; that curved tubulin was GTP-bound and straight tubulin was GDP-bound (Melki et al., 1989). This model was supported by early electron cryomicroscopy work that indicated that depolymerizing microtubules, in which the heterodimers at the plus-end are GDP-bound, have splayed protofilaments, while GTP-capped polymerizing microtubules have straight ends (Mandelkow et al., 1991). However, more recent evidence indicates that the curvature of the free heterodimer is not appreciably influenced by the nucleotide state (Chrétien et al., 1995; Buey et al., 2006; Rice Luke et al., 2008; Nawrotek et al., 2011; Ayaz et al., 2012; Pecqueur et al., 2012). Additionally, other works have identified long, curving protofilament bundles at the plus-ends of polymerizing microtubules (Chrétien et al., 1995; Müller-Reichert et al., 1998; Vitre et al., 2008; Guesdon et al., 2016). More recent electron cryotomography work using microtubules assembled from purified tubulin *in vitro*, as well as microtubules in cells, reveals flared individual protofilaments at polymerizing microtubules plus-ends (McIntosh et al., 2018; Figure 1C). However, these pieces of work do not explicitly test whether straightening the heterodimer directly impacts nucleotide state. While the plus-end of the growing microtubule continues to be an active area of investigation, together these works indicate that the switch between curved and straight conformations is nucleotide-independent and may represent a separate point of microtubule regulation.

The curvature of the tubulin heterodimer plays a critical role in microtubule polymerization and depolymerization at plus ends because it facilitates the formation and breaking of interactions between tubulin heterodimers. The connections between heterodimers within the microtubule are critical in facilitating microtubule dynamics. When a free tubulin heterodimer lands at the plus end of a protofilament, it establishes longitudinal interactions with the previously added heterodimer. These longitudinal interactions occur at the interdimer interface and are established between the T7 loop and helix 8 (H8) of the incoming α-tubulin and the T3 and T5 loops of the β subunit at the microtubule plus end (Löwe et al., 2001; Figures 1A,B). Along with longitudinal interactions, the tubulin heterodimer also forms lateral interactions with heterodimers in neighboring protofilaments. On both α- and β-tubulin, the lateral contacts are mediated by H1, sheet 2 (S2), H2, and S3 on one subunit, and the M loop on the α or β subunit on the adjacent protofilament (Löwe et al., 2001; Figures 1A,B). Importantly, curved protofilaments that splay out from the microtubule plus end can only support longitudinal interactions, while straight protofilaments in the lattice support both longitudinal and lateral interactions. Establishment of these interactions facilitates the straightening of the tubulin heterodimer, making them a crucial intrinsic control in microtubule dynamics.

These features of the tubulin heterodimer reveal the dynamic instability properties that are unique to microtubules, and importantly, distinct from actin and intermediate filaments mentioned above. Additionally, each of these properties serves as a potential point of microtubule regulation. Indeed, there are a multitude of proteins in cells that bind and regulate microtubules to perform specific cellular functions. This next section will broadly discuss the role of a few key microtubule motors and MAPs. We will then focus on the importance of these intrinsic and extrinsic regulators in neurodevelopment.

### Microtubule-associated proteins and motors

Broadly, MAPs encompass the group of proteins that physically interact with microtubules. MAPs are further classified into subgroups depending on function and activity. This review will not fully capture the multitude and complexity of all MAPs, instead we will briefly describe a few key players that are highly involved in microtubule dynamics so that we may further investigate how these MAPs influence microtubule networks during neurodevelopment. For a more comprehensive review on MAPs, we point the reader to different reviews covering this topic (Goodson and Jonasson, 2018; Bodakuntla et al., 2019).

One of the key MAP subclasses is microtubule plus-end tracking proteins, also known as “+ TIPS.” As the name suggests, this group is comprised of MAPs that selectively associate with microtubule plus-ends, rather than along the microtubule lattice. +TIPs include proteins that play important roles in regulating microtubule dynamics, such as proteins in the XMAP215 and EB families. While each of these proteins reside at plus ends, they each bind to different tubulin structures and ultimately have vastly different functions. XMAP215 is a processive, concentration-dependent microtubule polymerase (Brouhard et al., 2008). Polymerase activity involves specific TOG (tumor-overexpressed gene) domains that are found in all XMAP215 proteins and recognize the curved conformation of tubulin (Ayaz et al., 2012; Al-Bassam et al., 2012; Byrnes and Slep, 2017). This unique conformation-preference enables TOG domains to preferentially bind to free tubulin heterodimers and to heterodimers at the distal tips of protofilaments (Ayaz et al., 2012; Al-Bassam et al., 2012; Byrnes and Slep, 2017; Figure 1C). EB1 is the most well-studied member of the EB protein family and regulates microtubule dynamics by increasing polymerization rates, catastrophe frequencies, and rescue frequencies of microtubules *in vitro* (Vitre et al., 2008). While EB1 does increase polymerization rates, its impact is rather mild compared to the effect of XMAP215 on microtubule polymerization. While XMAP215 activity is modulated by the curved tubulin conformation, EB1 is sensitive to the nucleotide state. Specifically, EB1 binds the microtubule lattice at the junction of four tubulin heterodimers that are in a transition state following GTP hydrolysis (Maurer et al., 2012). Importantly, MAPs do not act alone in cells, rather they may work with or against one another to regulate microtubules. To this end, when XMAP215 and EB1 are combined *in vitro* they synergistically increase microtubule polymerization rates beyond what either protein does on its own (Zanic et al., 2013). These +TIPS and others form a complex network of proteins that regulate microtubule dynamics to promote the formation, maintenance, or disassembly of microtubules at the right place and time.

Another subgroup of MAPs is microtubule motors, which are classified as either kinesins or dyneins (Figure 1C). Kinesins are a vast group of ATPases that are comprised of 44 individual kinesin genes divided into 16 subfamilies in humans (Miki et al., 2001, reviewed in Kalantari and Filges, 2020). Kinesins hydrolyze ATP to propel predominantly plus-end directed movement along the external surface of the microtubule. Kinesins are canonically considered to transport cargo along microtubule tracks, however certain kinesins also influence microtubule dynamics. For example, kinesin-5 serves as a microtubule polymerase (Chen and Hancock, 2015) and kinesin-8 and kinesin-13 motors serve as microtubule depolymerases (Desai et al., 1999; Hunter et al., 2003; Varga et al., 2006; Varga et al., 2009; Friel and Howard, 2011). Dynein is also an ATPase-dependent motor, however unlike kinesins, it primarily moves toward the microtubule minus ends. Additionally, while there are 44 different kinesin genes in humans, there is only one cytoplasmic dynein. Dynein activity is highly regulated by a suite of accessory chains, adaptors and MAPs that influence complex stability, speed, processivity, as well as selective binding to the various cargoes it transports (Reck-Peterson et al., 2018). Beyond cargo transport, dynein also plays crucial roles in cell division and migration (Dantas et al., 2016). Particularly important in neuronal migration, dynein generates pulling forces on the microtubule cage surrounding the nucleus and centrosome to ensure they are moved toward the leading process. Additionally, dynein can regulate microtubule dynamics by destabilizing microtubules (Laan et al., 2012; Estrem et al., 2017). Together, kinesin and dynein motors rely on microtubules to deliver cargo throughout the cell. However, they also serve important roles in regulating microtubule dynamics either directly, such as kinesin-5 or -8, or indirectly by influencing the binding of other MAPs to the microtubule. We will further explore the roles MAPs play in regulating microtubule dynamics, and how they interface with intrinsic tubulin properties, in the final section of this review.

In the next section we dive into how MAPs influence microtubule dynamics during neurodevelopment. Additionally, we describe the consequences of altering these extrinsic regulators during the critical time period of neurodevelopment and consider what disease-mutants can teach us about the requirements for proper brain development.

## Microtubule regulation in neurodevelopment

### Microtubule networks during neurodevelopment

**FIGURE 2:**
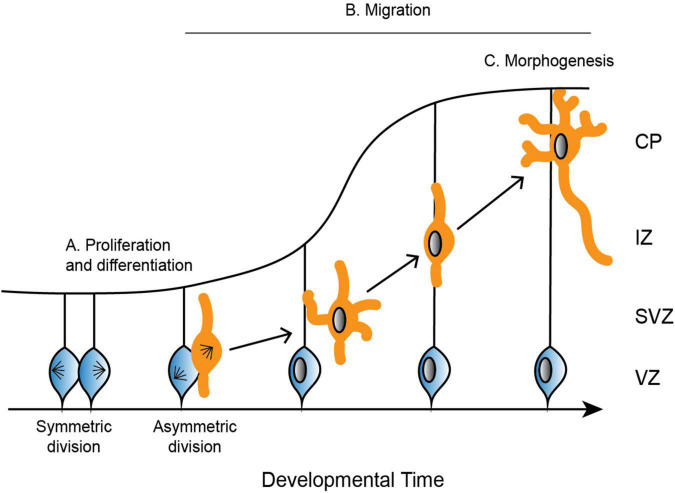
Depiction of the development of the cerebral cortex. **(A)** Neural progenitor cells (blue) undergo symmetric and asymmetric divisions before differentiating into specific cell populations, such as neurons (orange). **(B)** Neurons radially migrate along glial cells from the ventricular zone (VZ) to the cortical plate (CP). Neurons are bipolar in the VZ before switching to a multipolar state in the subventricular zone (SVZ). To continue radial migration, neurons then revert back to a bipolar state through the intermediate zone (IZ) until they reach the developing CP. **(C)** Upon reaching the CP, neurons undergo further morphogenesis, including maturation of the axon and extension of multiple dendrites.

For proper neurodevelopment, the microtubule cytoskeleton plays a critical role in major cellular events, including progenitor proliferation, neuronal migration, and neuronal morphogenesis (Figure 2; reviewed in: Kriegstein and Noctor, 2004; Götz and Huttner, 2005). Microtubules are not simply structural scaffolds in these processes; they are actively involved in cargo transport, cell signaling, and dictating highly dynamic processes like symmetry breaking. While there are many factors that influence neurodevelopment, we will focus here on the importance of microtubules and their associated proteins in this process, as well as examples of diseases that occur as a result of mutations in MAPs and tubulin. Understanding microtubule activity and regulation at each step in the process of neurodevelopment is crucial for our understanding of proper neurodevelopment, and how it can go awry when these regulatory steps are perturbed.

#### Proliferation and differentiation

In mammals, neurons are produced through a series of asymmetric and symmetric neural progenitor cell divisions (Figure 2A; Arai and Taverna, 2017). The differentiation of these neural progenitor cells is crucial for the specification of highly specialized cells that go on to form distinct structures of the brain. Defects in neural progenitor division can be catastrophic in either direction, as too many cell divisions may result in improper cellular differentiation, while delayed proliferation results in an under-production of specialized cell types. Aberrant divisions may give rise to incorrect cell types, or they may result in too many or too few progenitor cells at critical neurodevelopment time points.

Proliferation and differentiation require that progenitor cells form a bipolar mitotic spindle comprised of microtubules and an array of MAPs. Kinetochore microtubules capture and align chromosomes along the metaphase plate, and the chromosomes are separated by the force generated by the microtubules. This microtubule-generated force is highly regulated by a suite of microtubule motors and MAPs and relies on the coordination between many proteins to ensure proper chromosome segregation.

#### Migration

Following the differentiation of neural progenitor cells into neurons, different neuronal cell types migrate to specific regions of the developing brain (Reviewed in Marín and Rubenstein, 2003). Excitatory neurons radially migrate to the cortex and hippocampus, while inhibitory neurons tangentially migrate to the dorsal forebrain (Rakic, 1972; Altman and Bayer, 1990, reviewed in Kriegstein and Noctor, 2004). Excitatory neurons rely on multiple modes of transportation during radial migration, each of which requires dynamic and adaptable microtubule networks to remodel neuronal morphology. Newborn neurons undergo a brief period of bipolar locomotion in the ventricular zone before they move into the intermediate/subventricular zone and adopt a multipolar morphology with multiple dynamic processes (Figure 2B; Tabata and Nakajima, 2003). Neurons then transition back from a multipolar to a bipolar state. Axons are initiated during the multipolar stage and ultimately become the trailing process behind the stabilized pial surface-directed leading edge. Neurons then once again follow radial glial-guided locomotion until contacting the cortical surface and undergoing the terminal somal translocation, during which the apical dendrite is anchored to the marginal zone and the neuron detaches from the radial glia (Nadarajah et al., 2001; Santana and Marzolo, 2017).

The microtubule cytoskeleton is crucial throughout these stages of radial migration. During neuronal morphogenesis from bipolar to multipolar and back, microtubule dynamics are regulated via a complex signaling network (reviewed in Heng et al., 2010). Signaling pathways such as CDK5-driven phosphorylation of neuronal MAPs are crucial for adjusting the affinity, and thus the activity, between these MAPs and microtubules (reviewed in Heng et al., 2010). This tight regulation involving many MAPs are crucial in supporting the various process extension and retraction events that must occur during specific developmental time points. In the process of locomotion, microtubules are necessary to form a cage-like structure around the nucleus (Rivas and Hatten, 1995). The force generated to move the nucleus up the radial path to the cortical plate relies on dynein, as well as kinesins such as KIF1A and KIF2A (Homma et al., 2003; Tsai et al., 2007, 2010). Dynein, along with its regulator Lis1, generate the forces required to pull the nucleus and centrosome up the migrating cell toward the leading process (Tsai et al., 2007). Ultimately, each of these steps in neuronal migration requires properly regulated microtubules that respond to environmental signals to support the extension and retraction of processes, cargo transport, and signal transduction.

#### Morphogenesis

Once neurons have migrated to the upper cortical plate, they undergo further morphogenesis and differentiation as they mature the axon and extend many dendrites (Figure 2C). These elaborate morphologies are crucial for both sending signals through the axon, as well as receiving signals that are passed between thousands of other cells through the dendrites. Establishing neuronal polarity is therefore critical for proper brain function, and it is a complex process that relies on multiple intrinsic cellular and extrinsic environmental cues, many of which converge on the regulation of the microtubule cytoskeleton.

Neuronal polarity is established in part through microtubules. One of the defining distinctions in neuronal polarity is the plus-end out orientation of microtubules in the axon (Baas et al., 1988). In contrast, dendrites have mixed end microtubule polarity, meaning that a proportion of microtubules are plus-end out, while others are minus-end out (Baas et al., 1988; Burton, 1988). These differences in microtubule polarity have significant ramifications for cargo transport. Kinesins and dynein drive anterograde and retrograde transport, respectively, in axons (reviewed in Kevenaar and Hoogenraad, 2015). Dendrites on the other hand have dynein-driven anterograde transport (Kapitein et al., 2010). Microtubule polarity is a crucial part of proper cargo transport, which plays important roles in establishing proper neuronal polarity and morphology.

### Microtubules in neurodevelopment disorders

Much of our understanding of the importance of microtubule regulation in neurodevelopment stems from disease-associated mutations. For instance, mutations or deletions of MAPs, motors, or even tubulin itself results in neurodevelopment disorders. In this section we will discuss mutations in microtubule-related proteins that result in numerous neurodevelopment disorders, such as lissencephaly and pachygyria, polymicrogyria, and microcephaly. Each of these disorders present in patients with alterations to the folds (gyri) and grooves (sulci) that comprise the cortical structure. Lissencephaly is often referred to as “smooth brain” because patients have a total loss of folding patterns. Pachygyria is a milder version of lissencephaly as it still has folds and grooves, but the area of the folds is much broader, and the grooves are shallower. On the opposite end of the scale, polymicrogyria is characterized by having many gyri and sulci, and the folds are comparatively very small. Microcephaly is represented in patients with a smaller than typical brain. While each of these disorders are distinct, a single patient can present with a combination of these phenotypes, along with other neurological disorders not discussed here. This suggests that each of these disease-associated mutations may have multiple and nuanced effects on microtubule regulation, resulting in complex neurological phenotypes. Adding to this complexity, we know of some examples where mutations in different microtubule-related genes can give rise to similar phenotypes, but in other cases different mutations in the same gene are associated with vastly different disease states.

On the surface, this presents an interesting conundrum because cortical malformations cannot so easily be predicted from knowing the affected gene or location of the mutation within that gene. However, by examining what we know about microtubule regulation during neurodevelopment with what we know from in-depth studies of individual mutations, we may gain some understanding of mechanistic themes that link mutations to malformations. In this section, we highlight key mutations in microtubule-related proteins that result in one or multiple of these neurodevelopment disorders and discuss how studying these mutants can contribute to our understanding of proper neurodevelopment.

#### Microtubule-associated proteins in neurodevelopment

Studying the effect of mutations in MAPs that are linked with patient cortical malformations is important for several reasons when discussing the molecular mechanisms of tubulinopathies. The first reason is that studying MAP mutations in a disease context can provide great insight into how microtubules and microtubule regulators normally function in healthy contexts. Secondly, tubulinopathy mutations may lead to a loss of MAP binding and function, and therefore the tubulinopathy mutation may phenocopy mutations in MAPs that hinder their ability to properly regulate microtubule dynamics. And finally, some tubulinopathy mutations may alter the intrinsic properties of tubulin in a way that mimics constitutive MAP activity, ultimately subverting important local and temporal regulation that is normally provided by MAPs.

The first MAP to be specifically linked to lissencephalies is the gene lissencephaly 1 (LIS1) (Reiner et al., 1993). A homozygous *Lis1* knockout in mice is lethal, and heterozygous *Lis1*-null mice have disorganized cortical layers, among other neurodevelopment defects (Hirotsune et al., 1998). LIS1 is typically studied in the brain and testes, but Lis1 mRNA is expressed in many murine cell types, and different cell types express various splice variants (Péterfy et al., 1998). In migrating neurons, LIS1 localizes primarily to the centrosome, and depletion of LIS1 in mice results in delayed and/or reduced neuronal migration (Hirotsune et al., 1998; Gambello et al., 2003; Tanaka et al., 2004). Importantly, LIS1 interacts and co-localizes with dynein and dynactin, and together these proteins pull the nucleus and centrosome toward the leading process during neuronal radial migration (Smith et al., 2000; Faulkner et al., 2000; Tsai et al., 2007). Additionally, LIS1, dynein, and dynactin localize to the growth cone and promote axon extension (Grabham et al., 2007). Depletion of LIS1 or dynein disrupts nuclear movement, further emphasizing the importance of these two proteins working together during neuronal migration (Shu et al., 2004; Tsai et al., 2007). Furthermore, LIS1 interacts directly with microtubules as well as tubulin heterodimers and reduces the frequency of microtubule catastrophe events *in vitro* (Sapir et al., 1997). Patient mutations have been identified across the LIS1 gene with no evident correlation between location within the gene and degree of lissencephaly severity observed in patients (Saillour et al., 2009). Furthering our understanding of how specific residue changes in LIS1 impact microtubules on a molecular level will be insightful in understanding changes observed in neuronal migration and ultimately tissue development.

A second critical MAP linked to lissencephaly is the protein doublecortin (DCX) (des Portes et al., 1998; Gleeson et al., 1998). *DCX* is located on the X chromosome and is specifically expressed during the proliferation of neuroprogenitors, and later during differentiation and neuronal migration (Francis et al., 1999; Gleeson et al., 1999; Kappeler et al., 2006; Koizumi et al., 2006). Its chromosome localization makes it a predominantly male-associated disease, though heterozygous female patients that have been identified have a milder, more mosaic phenotype. The DCX protein is localized at the leading edge of migrating neurons, consistent with interneurons derived from *DCX* knockout mice forming excessive branches with very short lifetimes (Friocourt et al., 2003; Kappeler et al., 2006). Additionally, these interneurons have disorganized migration patterns (Kappeler et al., 2006). DCX plays a crucial role in microtubule stabilization by promoting nucleation of 13 protofilament microtubules, as well as cooperatively binding and tracking of these 13 protofilament plus-ends (Moores et al., 2004; Bechstedt and Brouhard, 2012). Disease-associated mutations in DCX are found across the gene, with no obvious clustering in a particular domain that gives immediate insight into the effect of these mutations on microtubules and neurodevelopment (Bahi-Buisson et al., 2013). Presumably these mutations alter microtubule stability and/or binding of other MAPs to the microtubule. There has been substantial work uncovering the role of DCX in migrating neurons and ultimately neurodevelopment, however questions remain regarding how individual DCX residue positions and amino acid substitutions determine malformation severity (Reviewed in Bahi-Buisson et al., 2013; Ayanlaja et al., 2017).

#### Motors in neurodevelopment

Along with mutations identified in classical MAPs that are linked to neurodevelopment disorders, there have also been numerous cases linking mutations in microtubule motors to these disease states. Dynein cytoplasmic 1 heavy chain 1 (*DYNC1H1*), in this review referred to as dynein, is a minus-end directed motor that is expressed in a majority of human tissues. Patients harboring heterozygous mutations in dynein often present with polymicrogyria, which is identified as having many cortical folds that are abnormally small (Willemsen et al., 2012; Poirier et al., 2013; Fiorillo et al., 2014; Laquerriere et al., 2017; Amabile et al., 2020). Despite the functional intersection between dynein and LIS1, patient mutations in LIS1 are associated with lissencephaly, while dynein mutants are more often associated with polymicrogyria. Therefore, additional work in mouse models has been done to elucidate the role of dynein in neuron migration and why these patient mutations may result in unique patient phenotypes. Mice that are homozygous null for dynein are inviable, while heterozygous mice have no distinguishable defects (Harada et al., 1998). This suggests that one functional copy of dynein is sufficient for proper neurodevelopment in mice. Considering dynein is reliant on multiple regulators for proper function, it would be reasonable to consider that these regulators may help compensate for the depleted dynein levels in the heterozygous mice. Beyond its role in generating pulling forces on the nucleus and centrosome during neuronal migration, dynein serves as the primary retrograde motor for axonal transport, due to axonal microtubules being plus-end out (Heidemann et al., 1981; Baas et al., 1988). Dynein motors are necessary for plus-end out microtubule orientation, as well as trafficking specific cargos from the distal tip of the axon to the cell body (Zheng et al., 2008; Satoh et al., 2008; reviewed in Hirokawa et al., 2010). Multiple groups have thus used ENU-induced heterozygous missense mutations or RNAi depletion to study the effects of dynein in neurodevelopment and neurodegeneration. In these models, neuronal migration was perturbed, and dynein-directed retrograde axonal transport of key cargoes were delayed and, in some instances, significantly impaired (Hafezparast et al., 2003; Ori-McKenney and Vallee, 2011; Zhao et al., 2016). Interestingly, patient mutations have been identified across *DYNC1H1*, with varying degrees of patient phenotypes and associated severities (Harms et al., 2012; Hoang et al., 2017; Beecroft et al., 2017). However, patients that present with cortical malformations typically have mutations clustered in the motor domains (Harms et al., 2012; Hoang et al., 2017). It has been speculated that some of the mutants may interfere with destabilizing the conformational state of dynein during ATP hydrolysis (Hoang et al., 2017). Further work on multiple patient-associated dynein mutants located across the protein indicate that these mutations have many different effects, including in some cases defects in spindle positioning and altered motility *in vitro* (Marzo et al., 2019). Studying disease mutants, as well as studying the role of different dynein domains, played a crucial role in advancing our understanding of how patient mutations that occur throughout the protein contribute to impaired neurodevelopment (Niekamp et al., 2019). Applying ideas and studies such as these to tubulinopathy mutations may be crucially important to developing mechanistic themes about how tubulinopathies arise.

While dynein serves as the primary minus-end directed motor, the kinesin families principally function as plus-end directed motors. Similar to dynein, there are several mutations that have been identified in different kinesins that are associated with neurodevelopment disorders. In humans, there are 44 identified kinesin family member genes, which are further split into 16 subfamilies (Miki et al., 2001; Kalantari and Filges, 2020). While mutations in many kinesins are associated with neurological disorders, we focus here on three specific kinesins that have been associated with cortical malformations: KIF5C, KIF2A, and KIF21B.

KIF5C is a member of the kinesin-1 family and is particularly enriched in neuronal cells (Kanai et al., 2000). Patient mutations that have been identified in KIF5C have presented as pachygyria, characterized by broad folds and shallow grooves (Poirier et al., 2013; Willemsen et al., 2014; Jamuar et al., 2014; Cavallin et al., 2016; Michels et al., 2017). To date, five of the six KIF5C mutations identified occur at the glutamate residue 237, while the sixth mutation is located at alanine residue 268. Both residues reside in the microtubule-binding domain of KIF5C, which functional and structural studies have indicated is necessary for selective accumulation of KIF5C in the axon (Nakata et al., 2011; Morikawa et al., 2015). In accordance with these data, mutating glutamate 237 to a valine inhibits ATP hydrolysis and disrupts KIF5C localization at the cell cortex (Poirier et al., 2013). To date, the molecular impact of the other KIF5C mutations is unknown, however it is reasonable to consider that other mutants identified in the motor domain may have similar effects. Further work addressing the neuronal cellular impact of improper KIF5C localization and activity will further elucidate how these mutations result in cortical malformations such as pachygyria.

Another kinesin that has been identified to have mutations linked to neurodevelopment disorders is KIF2A, a member of the kinesin-13 family, which is expressed throughout human tissues. Patient phenotypes from different KIF2A mutants present as lissencephaly, pachygyria, and microcephaly, or a combination of these malformations (Poirier et al., 2013; Tian et al., 2016; Cavallin et al., 2016). In-depth studies of two of these mutants reveal that ectopic expression of disease-causing mutants disrupt interneuron migration, as well as the proliferation of progenitor cells (Broix et al., 2018). In developing neurons, KIF2A is localized to growth cones of developing axons and plays critical roles in axonal branching and pruning (Noda et al., 1995; Homma et al., 2003; Maor-Nof et al., 2013). Similar to the mutations identified in KIF5C, all mutations in KIF2A reported to date are located in the same region. However, in the case of KIF2A mutants, all are located in the motor domain (Poirier et al., 2013; Tian et al., 2016; Cavallin et al., 2016; Broix et al., 2018). KIF2A functions as a microtubule depolymerase, specifically by inducing a destabilizing conformational change at microtubule ends (Desai et al., 1999). Microtubule regulation via KIF2A’s microtubule depolymerase activity is clearly linked to proper cortical development, however future work will need to determine precisely when and where KIF2A activity is required.

KIF21B is a member of the kinesin-4 family and is predominantly expressed in the human testes, spleen, and is highly enriched in neurons (Marszalek et al., 1999). Mice that are homozygous null for KIF21B display microcephaly and isolated neuronal cultures have decreased dendritic complexity (Muhia et al., 2016; Kannan et al., 2017). To date, three patients have been identified with missense mutations in KIF21B. Interestingly, each patient presents with a different brain structure; one patient presents with microcephaly, one with complete agenesis of the corpus callosum, and the third with no notable brain structure abnormalities (Asselin et al., 2020). Further analysis into these three patient-associated mutations highlight that, to varying degrees, the KIF21B mutants impair neuronal migration by increasing KIF21B motor activity (Asselin et al., 2020). The results of recent molecular studies of KIF21B are complex. For example, the depletion of KIF21B by RNA interference in neuronal cultures results in increased microtubule polymerization rates (Ghiretti et al., 2016), but a full KIF21B knockout results in decreased rates (Muhia et al., 2016). However, *in vitro* work indicates that full-length KIF21B increases microtubule growth rates (Ghiretti et al., 2016), while other work indicates that the KIF21B motor domain alone decreases growth rates *in vitro* (van Riel et al., 2017). These seemingly contradictory pieces of work highlight the importance of understanding the contribution of individual protein domains and residues in regulating microtubule dynamics and cargo transport, and the ultimate effect of this on neurodevelopment.

Patient-associated mutations identified in both MAPs and motors have been crucial to our understanding of the important mechanisms that underlie proper neurodevelopment. The focus of the next section will be centered around patient-associated mutations in tubulin that are associated with neurodevelopment disorders, and whether the field can predict the molecular and tissue-level outcomes of this multitude of mutants.

## Tubulinopathies

Advancements in patient exome and whole genome sequencing have enabled the identification of single nucleotide polymorphisms (SNPs) linked to cortical malformation disorders. To date, mutations have been identified in four human β-tubulin isotypes and one of the α-tubulin isotypes (Bahi-Buisson et al., 2014; Maillard et al., 2022). While a subset of mutations leads to particular patient cortical malformations, other mutations, often in the same gene or even domain, result in other types of malformations. In this section, we discuss whether certain phenotypes can be attributed to the expression levels of certain isotypes during development. We also describe specific tubulin mutations and how those relate to the patient-observed cortical malformations. Whether these changes can be attributed to the type of change in amino acid, or if it is residue/domain specific, remains a large and unanswered question in the field.

### Tubulin during neurodevelopment

#### Tubulin expression

Humans and many other species express multiple α- and β-tubulin genes, known as isotypes. Isotypes are differentially expressed according to cell type and developmental timing, and encode proteins that exhibit high levels of conservation, but an array of subtle sequence differences (Ludueña and Banerjee, 2008). A majority of these sequence differences exist in the C-terminal tails, the 10–20 amino acids at the carboxy-terminus of α- or β-tubulin. In some cases, these sequence differences have been shown to impart significant functional differences (Sirajuddin et al., 2014; Vemu et al., 2017). These different isotypes can co-polymerize together, forming diverse microtubules composed of multiple tubulin isotypes (Lewis et al., 1987). While the isotypes have the capacity to co-polymerize with one another, the varying expression levels of the isotypes in different cell types and throughout development indicate that not all isotypes may be involved in all cellular functions, at all times. Therefore, it remains an open question whether isotypes show selective enrichment into particular subsets of microtubules. Studying the expression and role of various isotypes in neurodevelopment may provide great insight into the importance of varied tubulin expression in neurodevelopment and disease.

A classic example of unique cell type and developmental time point expression of tubulin isotypes is the β-tubulin-III (*TUBB3*). In the brain, *TUBB3* is classified as a neuron-specific β-isotype (Jiang and Oblinger, 1992; Burgoyne et al., 1988). Additionally, *TUBB3* expression is high in neurons both in the fetus and at birth, then decreases throughout postnatal development (Denoulet et al., 1986; Jiang and Oblinger, 1992; Miller et al., 2014). Interestingly, despite being a neuron-specific isotype, *Tubb3-/-* mice have no discernible neurodevelopment defects or cortical malformations (Latremoliere et al., 2018). In neurons from the *Tubb3* knockout mice there is a decrease in microtubule polymerization rates in growth cones, as well as a decrease in neurite outgrowth (Latremoliere et al., 2018). Importantly, there is an increase in other β-tubulin isotypes so that total β levels are unchanged, indicating that other isotypes are upregulated to compensate for the loss of *Tubb3* (Latremoliere et al., 2018). Together, these data indicate that while *TUBB3* is a neuron-specific isotype, it is not required to maintain the appropriate amount of total β-tubulin, nor is it required for proper neurodevelopment. However, the observed changes in microtubule polymerization rates are interesting and are consistent with different isotypes exhibiting various microtubule dynamics, and that isotypes co-polymerizing in different proportions are likely to alter dynamics (Vemu et al., 2017).

It is interesting to note that despite *Tubb3-/-* mice displaying relatively normal neurodevelopment, specific *Tubb3* point mutations disrupt proper brain development in mice (Tischfield et al., 2010). There are a number of patient cases that have heterozygous, missense mutations in *TUBB3* and present with severe neurodevelopment phenotypes (Poirier et al., 2010; Oegema et al., 2015; Park et al., 2021). This suggests that either point mutations act in a dominant manner that is distinct from the total loss of *TUBB3*, or that the requirement for TUBB3 function is higher for human brain development compared to mouse brain development. These disease-associated mutations have a range of clinical phenotypes; some have cortical malformations such as polymicrogyria, while others have a relatively well-developed cortex but display agenesis or hypoplasia of the corpus callosum or basal ganglia dysplasia (Poirier et al., 2010; Oegema et al., 2015; Park et al., 2021). Additionally, these mutants form heterodimer and incorporate into microtubules at various efficiencies (Poirier et al., 2010; Oegema et al., 2015). Comparing phenotypes between *Tubb3-/-* mice and *TUBB3*-tubulinopathy mutations indicates that proper neurodevelopment is achieved in animals completely lacking *Tubb3*, but not in animals or patients that harbor a missense mutation in the gene. Clearly the cells can compensate in the absence of *Tubb3* with other β-isotypes, indicating that these *TUBB3*-associated tubulinopathies are acting in a different mechanism that cannot be solely explained by changes in isotype expression due to haploinsufficiency. Based on RNA-seq data gathered from the whole fetal brain, *TUBB3* makes up approximately 17% of β-tubulin at this crucial developmental time point (Miller et al., 2014; Park et al., 2021). Therefore, how missense mutations in a β-tubulin isotype that represents only a portion of the tubulin pool can exert dominant effects on neurodevelopment will be critically important in future studies.

On the other side of the heterodimer is α-tubulin, which similarly to β-tubulin, has multiple isotypes in humans and other vertebrates, and these isotypes are expressed at various levels in different cell types and points during development (Ludueña and Banerjee, 2008). The most highly expressed α-tubulin isotype in post-mitotic developing neurons is *TUBA1A* (Miller et al., 1987; Gloster et al., 1994; Gloster et al., 1999). Unlike *Tubb3*, *Tuba1a* is seemingly indispensable in mouse neurodevelopment and *Tuba1a*-/- is lethal (Bittermann et al., 2019). Interestingly, there is only a slight reduction in survivability in mice that have only one copy of *Tuba1a* deleted (Bittermann et al., 2019). Analysis of the fetal forebrain indicates that *Tuba1a-/-* mice have an increase in ventricular zone width, and decreased intermediate zone and cortical plate widths, as compared to WT and *Tuba1a* heterozygous mice (Bittermann et al., 2019). Mice do not develop gyri and sulci as humans do, and therefore their brains are always smooth, making it difficult to do a side-to-side comparison with human cortical malformations, such as lissencephaly. However, the changes observed in the developing cortex regions in the *Tuba1a-/-* mice is consistent with neuronal migration defects that result in cortical malformations in patients (Keays et al., 2007). Compared to total protein levels at this developmental time point, total α-tubulin is decreased in brain lysates of *Tuba1a-/-* mice compared to WT (Bittermann et al., 2019). This indicates that for Tuba1a, other α-isotypes are not sufficiently upregulated in response to the knockout. This suggests that the requirement for *TUBA1A* in the human developing brain may be quite distinct from *TUBB3*.

Results from unbiased, ENU mutagenesis screens conducted in two labs further demonstrate the unique requirements of *Tuba1a* in mouse neurodevelopment. One study identified a *Tuba1a*-S140G mutant that disrupts neuronal radial migration, a key feature of many cortical malformations (Keays et al., 2007; Belvindrah et al., 2017). More recently, an analogous mutation in human *TUBA1A* has been identified in a patient that is documented to display microcephaly and aplasia/hypoplasia of the corpus callosum (from DECIPHER database, ref ID: 273321; Hebebrand et al., 2019). Further analysis reveals that *Tuba1a*-S140G has reduced GTP-binding and heterodimer formation, although the mutant heterodimer is still able to incorporate into microtubules (Keays et al., 2007). Summarizing these data, *Tuba1a-/-* and *Tuba1a*-S140G heterozygous mice both have impaired neuronal migration (Keays et al., 2007; Belvindrah et al., 2017; Bittermann et al., 2019), while *Tuba1a+/-* mice have no migration defects (Bittermann et al., 2019). These data cannot be explained simply by a reduction in the amount of *Tuba1a* expression but may rather be explained by the S140G mutant having a dominant effect on the microtubules in which it incorporates, and ultimately brain development.

A second ENU-generated mutation to note is *Tuba1a*-N102D, which was identified in a forward genetic screen searching for locomotor defects in mice (Gartz Hanson et al., 2016). *Tuba1a^ND/ND^* mice are neonatal lethal, have small brains, and display defects in cortical layering in the cerebral cortex (Gartz Hanson et al., 2016). The heterozygous *Tuba1a^ND/+^* mice survive to adulthood, but do not form proper midline commissures (Buscaglia et al., 2022). Additionally, *Tuba1a^ND/+^* brains have significantly reduced levels of total α-tubulin at P0 (Buscaglia et al., 2020), and the mutant is unable to incorporate into microtubules in neuronal cultures (Buscaglia et al., 2022). At the molecular level, the N102D mutant disrupts heterodimer stability and incorporation into the microtubule lattice (Gartz Hanson et al., 2016). The N102D mutant is an interesting way to probe the importance of *TUBA1A* in the developing brain, particularly because it provides insight into the necessity of having not only sufficient *TUBA1A* protein levels, but also a level of *TUBA1A* function that is sufficient for microtubule dynamics, neuronal cell biology, and ultimately tissue formation.

It is clear from these two ENU-generated mouse models that *Tuba1a* plays a critical role in neurodevelopment, and that compensation for *Tuba1a-/-* by other α-isotypes does not occur in a similar manner as seen for *Tubb3*^–/–^. The molecular and phenotypic differences between the *Tuba1a*-S140G and *Tuba1a*-N102D heterozygous mice also point toward the possibility that neurodevelopment defects may have different molecular drivers, depending on specifics of the missense mutation such as what the mutation is, and where it is located. Therefore, it is interesting to probe further into *TUBA1A* specific tubulinopathy mutations and determine if there are particular patterns or themes that we can draw from these studies about how particular regions influence *TUBA1A* biology and ultimately neurodevelopment.

### Investigating specific residues affected by mutations in patients; can they tell us more about the etiology of tubulinopathies?

Much of the field’s understanding of how specific tubulinopathy-associated amino acid changes impact molecular and/or cellular function stem from a handful of studies of the mechanistic impact of a particular mutation. There are 119 heterozygous, missense mutations that have been identified in *TUBA1A* to date (Hebebrand et al., 2019). Therefore, while these individual studies can provide important insights into how a certain mutant alters microtubule dynamics and neuronal cell biology, it would be useful to identify mechanistic themes that apply across multiple of these mutations. Here, we summarize the work that has been done in these studies modeling patient mutations. Additionally, we attempt to identify themes that span across these studies in order to gain a greater understanding of how *TUBA1A* mutants impact molecular and cell biology that ultimately lead to cortical malformations.

The first study of a patient mutation, *TUBA1A*-R264C, suggested that the mutant may act through a haploinsufficiency mechanism. This mutant was first identified in a patient that presented with pachygyria (Keays et al., 2007). Further analysis revealed that the R264C mutation decreases the formation of α-/β-tubulin heterodimer *in vitro*, as a result of decreased affinity between the mutant α-tubulin and a tubulin chaperone, TBCB, that is important for tubulin heterodimer biogenesis (Tian et al., 2008). Despite the reduction in the biogenesis of heterodimers containing α-tubulin R264C, when a heterodimer is formed from this mutant tubulin it incorporates into microtubules, albeit at a reduced rate. Based on these results, the authors conclude that the decreased formation of heterodimer from α-tubulin R264C is consistent with a haploinsufficiency phenotype; essentially, there is insufficient functional heterodimer to support necessary microtubule structures and dynamics (Tian et al., 2008). While haploinsufficiency could be a cause of disease, it is important to note that this study does not address whether total α-tubulin levels are altered in neurons that express R264C. This is an important consideration, given the robust tubulin biogenesis pathways that produce heterodimers in cells. Furthermore, it would be important to investigate whether microtubules that do have R264C heterodimer incorporated have altered dynamics compared to microtubules containing wild-type tubulin. Answering questions such as these would provide the field valuable insight into whether these *TUBA1A* mutants have the capacity to dominantly disrupt microtubule dynamics and function, and to what extent cells may have the capacity to mitigate these effects.

Whether TUBA1A tubulinopathy mutants all act through a common mechanism of haploinsufficiency was called into question in 2010, when Tian et al. (2010) investigated nine additional tubulinopathy disease-associated mutants in *TUBA1A* [I188L, P263T, L286F, R402C, R402H, S419L, I238V, and L397P]. These mutants are spread across the *TUBA1A* gene with no apparent clustering that aligns with molecular or patient-level phenotype (Tian et al., 2010). The results of this study reveal that some mutants disrupt the tubulin heterodimer assembly pathway, while others are unstable *in vitro*, and others impact microtubule assembly (Tian et al., 2010). These results suggest that some mutants result in haploinsufficiency while others mutants may act dominantly to perturb microtubule dynamics. Understanding how these two different scenarios ultimately result in similar patient phenotypes is an outstanding question.

As stated above, it had been previously found that *TUBA1A*-R402C significantly, and -R402H slightly, reduces the amount heterodimer *in vitro* (Tian et al., 2010). However, they also find that these mutants do incorporate into microtubules (Tian et al., 2010). Although these mutants do not form heterodimer *in vitro*, it was later found that these mutants do form heterodimer in cells (Aiken et al., 2019). This study showed that *TUBA1A*-R402C and -R402H assemble into microtubule polymer and act dominantly to disrupt neuronal migration in mice (Aiken et al., 2019). Furthermore, when modeled in budding yeast, these mutations produce normal amounts of heterodimer that incorporates into microtubules. Strikingly, microtubules containing R402 mutant tubulins disrupt, but do not fully ablate, dynein activity (Aiken et al., 2019). This indicates an alternative model for tubulinopathies where if these mutant tubulins are able to assemble into microtubules, they may then alter the binding of MAPs.

Our recent work further highlights the importance of the interactions between tubulin subunits, MAPs, and the interplay between the two. Our recent investigation of *TUBA1A*-V409I and V409A highlights the ability of these mutants to polymerize into microtubules and dominantly disrupt neuronal migration and morphologies (Hoff et al., 2022). Similar to the above mentioned studies of R402C and R402H tubulinopathy mutants, the V409I and V409A mutants were modeled in cultured neurons and in budding yeast to investigate molecular and cellular-level effects. In this case, the two mutants disrupt the microtubule dynamics regulation conferred by XMAP215/Stu2, and the more severe V410A mutant intrinsically drives increased microtubule polymerization rates in *in vitro* reconstitution experiments with purified tubulin. These findings highlight the importance of studying points of both the intrinsic activity of tubulin proteins and extrinsic microtubule regulation by MAPs, and how these areas interact to control microtubule dynamics throughout development and disease.

**FIGURE 3:**
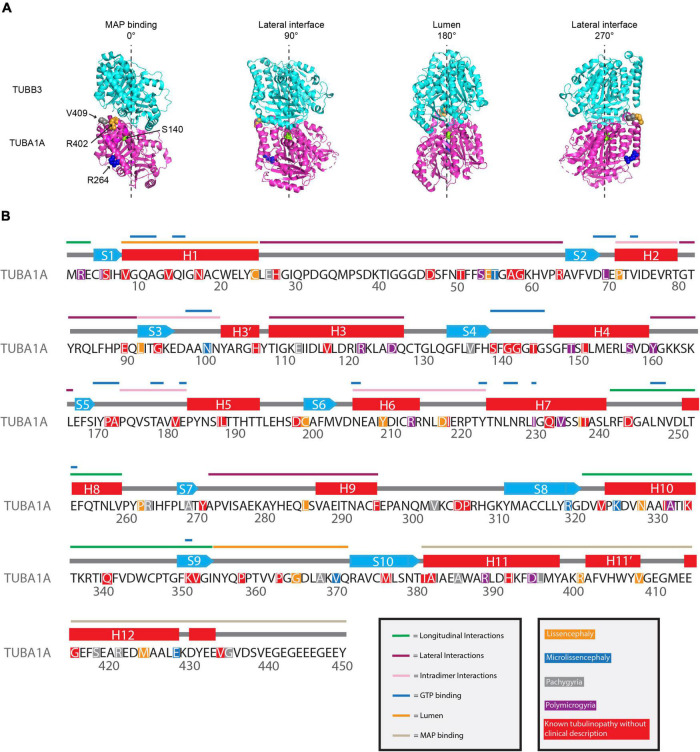
Mapping *TUBA1A* tubulinopathy mutations. **(A)** Mapping individual mutants described in section “Investigating specific residues affected by mutations in patients; can they tell us more about the etiology of tubulinopathies?” on TUBA1A (PDB structure: 5JCO). α-tubulin is human TUBA1A in magenta and β-tubulin is human TUBB3 in cyan. Spheres represent TUBA1A residues S140 (green), R264 (blue), R402 (orange), and V409 (gray). Four views of the structure are shown, each rotated 90° along the longitudinal axis. The 0° angle represents the outer surface of the microtubule that is considered the MAP binding region. 90° and 270° angles represent lateral interfaces with adjacent protofilaments. 180° represents the luminal side of the microtubule. **(B)** Known *TUBA1A* mutations to date mapped on primary sequence according to predominant cortical malformation. If no clinical imaging data is available to us, the mutation is highlighted in red. Functional regions described in section “Mapping the tubulinopathy mutations” are labeled in various colors above the secondary structures.

While these individual examples do not encompass all of the work that has been done to decipher how tubulinopathy mutations result in such catastrophic cortical malformations, they provide a sample of how the field has expanded its thinking of the mechanistic themes underlying these diseases. While some work has indicated that *TUBA1A* tubulinopathy mutations result in haploinsufficiency, more recent works have highlighted how patient mutations dispersed across the α-tubulin protein can act dominantly from within microtubules to alter dynamics and interactions with MAPs (Figure 3A). The ability of many of these mutant tubulins to incorporate into microtubules and dominantly disrupt everything from MAP binding, microtubule dynamics, neuronal cell biology, and tissue structures indicates that the field should be considering these mutations at multiple levels.

### Mapping the tubulinopathy mutations

The increasing number of identified tubulinopathy-associated mutations emphasizes the need to identify broader mechanistic themes that link molecular level tubulin defects to tissue level disease. In the long term, identifying these links will be useful for predicting the phenotypic outcome of specific mutations and identifying new treatment options. As mentioned previously, the tubulin heterodimer has multiple interfaces that are the intrinsic regulatory elements of microtubule dynamics. These interfaces include the nucleotide N- and E-sites, as well as longitudinal and lateral interfaces. Despite these regions being recognized as key intrinsic regulators of microtubule dynamics, many tubulinopathy mutations reside outside these regions and yet still impact microtubule activity.

Other regions are predicted and/or have been identified to interact with various MAPs and motors. These MAPs and motors have primarily been identified to interact with the external sides of the microtubule. It is important to note that another class of microtubule binders have been identified, known as microtubule inner proteins (MIPs), although to date this class of proteins have only been identified to interact with microtubule doublets that are present in the axonemes of cilia and flagella (Nicastro et al., 2006, 2011). This class of proteins is much less studied compared to MAPs and motors; however, it is important to consider that there are residues within the microtubule lumen that may interact with these MIPs. Identifying whether *TUBA1A* mutations may impact one or multiple of these interacting sites will be important in understanding the functional consequences of these mutants and may provide further insight into the specific roles of microtubules in neurodevelopment.

To understand if we can decipher patterns between *TUBA1A* mutations and disease phenotype and/or severity, we mapped the tubulinopathy mutants in several ways. From a list of the 119 *TUBA1A* mutations identified to date, we classified each mutation with the predominant cortical malformation that has previously been described in the literature (Hebebrand et al., 2019). The major cortical malformations we identified in the literature and that we used for the remainder of the analysis in this section are lissencephaly, microlissencephaly, pachygyria, and polymicrogyria. If no clinical assessment was available to our knowledge, we classified the mutation as “not available”. Since one might expect mutations with similar malformations to affect similar regions of the tubulin protein, we mapped the mutants along the primary sequence. However, the distribution of mutations does not reveal any striking enrichment of mutations that result in similar malformations anywhere along the primary amino acid sequence (Figure 3B). Rather, the mutations that cause lissencephaly, microlissencephaly, pachygyria, and polymicrogyria appear to be randomly distributed across the gene.

**FIGURE 4:**
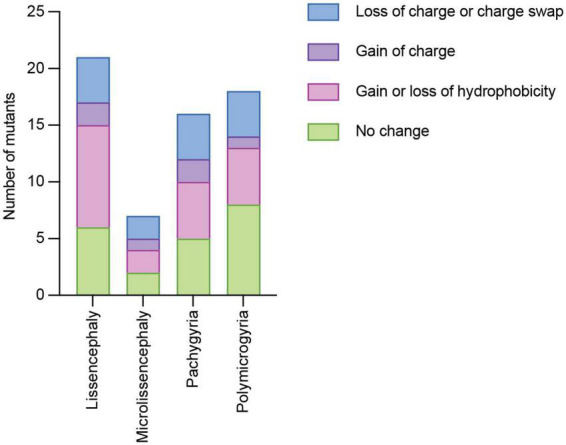
Quantifying *TUBA1A* mutations by type of amino acid side chain change. *TUBA1A* mutations were sorted by the type of amino acid change that occurs in patients (loss of charge or charge swap, gain of charge, gain or loss of hydrophobicity, or no change in charge or hydrophobicity). *TUBA1A* mutations were also sorted by the primary cortical malformation resulting from each mutation (lissencephaly, microlissencephaly, pachygyria, or polymicrogyria). For each cortical malformation, the number of mutations that qualify as one (or multiple) of these types of amino acid changes were reported.

The charge and hydrophobicity of amino acids are important for a multitude of protein functions Therefore, we considered whether different types of amino acid side chain changes were linked to specific cortical malformations. Of the 119 *TUBA1A* mutations identified, 59 of them do not have clinical neuroimaging data publicly available. Therefore, we excluded these mutations from the following analysis. We classified these types of amino acid changes as: (1) loss of charge or charge swap, (2) gain of charge, (3) loss or gain of hydrophobicity, and (4) no change in charge or hydrophobicity. Tubulin electrostatic charges are critical for interactions with MAPs and motors, therefore we reasoned that a loss of charge or a charge swap could have significant implications for microtubule interactors. On the other hand, a mutation that results in a gain of charge on a previously uncharged amino acid could potentially impact microtubule interactors, but it could likely have different effects depending on where in the protein it is located, and therefore we made a separate category. The loss or gain of hydrophobicity could have significant impacts on protein folding. The final classification of no change represents mutations that do not impact the charge or hydrophobicity from the original amino acid, calling into question how these seemingly benign mutations cause such devastating malformations. It is important to note that these categories are not all mutually exclusive. For example, one mutation could result in a loss of charge as well as a gain of hydrophobicity. We find that each malformation is associated with mutants with at least one of each type of amino acid change (Figure 4). Because these data are not mutually exclusive, we are unable to run statistics on the data set to determine whether the types of amino acid changes are significantly different than one another. However, because each malformation is associated with at least one mutant from each type of amino acid change, we asked whether we could identify common mechanisms of these mutants by categorizing by where the mutations lie within the protein structure.

**FIGURE 5:**
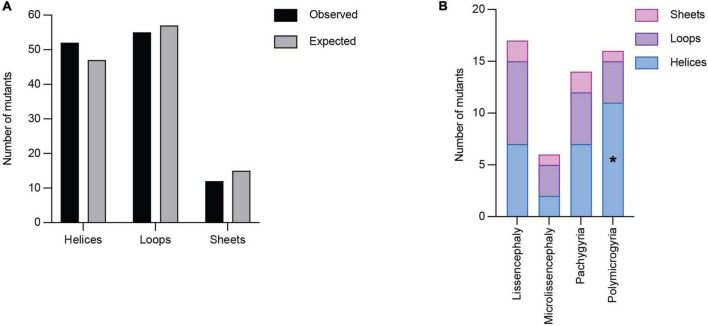
Quantifying *TUBA1A* mutations by secondary structure. **(A)** *TUBA1A* mutations were sorted by the secondary structural element (helix, loop, or sheet) in which they reside. Observed and expected number of mutations were compared using a Chi-squared test [5.99 critical value when α = 0.05; X^2^ (df = 2, *N* = 119) = 1.202; *p* = 0.55]. The expected number of mutations in each secondary structure was calculated by determining the percentage of amino acids that reside in each structure, then multiplying that by 119 (the number of *TUBA1A* missense mutations known to date). This value represents the number of mutations that would be expected to appear in each structure if all 119 mutations were randomly distributed. **(B)** *TUBA1A* mutations were sorted by the primary cortical malformation resulting from each mutation (lissencephaly, microlissencephaly, pachygyria, or polymicrogyria). For each cortical malformation, the number of mutations in each secondary structural element was reported. A Fisher’s exact test was run for each cortical malformation category to determine if mutations were enriched in one secondary structure over the others. Asterisk (*) on bar indicates *p*-value < 0.05 (polymicrogyria helices *p* = 0.03).

Secondary structures play crucial roles in protein folding, structure, and function. *TUBA1A* secondary structure consists of approximately 39% helices, 12% sheets, and 49% loops (Löwe et al., 2001). Using all 119 *TUBA1A* mutants, regardless of whether there is publicly available clinical data, we asked whether tubulinopathy mutations occur more frequently in a particular secondary structure, potentially revealing a bias for disease-associated mutations. Based on a Chi-squared test, we find there is no significant enrichment of *TUBA1A* mutations in any of the secondary structures beyond what would be expected if the mutations were randomly distributed [Figure 5A; 5.99 critical value when α = 0.05; X^2^ (df = 2, *N* = 119) = 1.202; *p* = 0.55]. This indicates that there is not a general enrichment of mutations in the secondary structures. We next asked whether mutations associated with a particular cortical malformation were biased toward particular secondary structures. Therefore, for this analysis we used only the 53 *TUBA1A* mutations that have available clinical neuroimaging data. For each individual malformation category, we used a Fisher’s exact test to determine whether the number of mutants found in one secondary structure were significantly different than would be expected if the mutations were random. We find that the majority of *TUBA1A* mutations are not significantly enriched in a particular secondary structure. However, the number of polymicrogyria mutations found in helices is significantly higher than what would randomly be expected (Figure 5B; Table 1; *p* = 0.03). This indicates that polymicrogyria mutations in *TUBA1A* are more likely to be found in helices. Further work will be needed to investigate the link between disrupting helical structures and the high number of small gyri observed in polymicrogyria patients.

**TABLE 1:** Fisher’s exact tests were performed to determine whether the associations between cortical malformation mutants and either secondary structures or functional domains are non-random.

		Lissencephaly	Microlissencephaly	Pachygyria	Polymicrogyria
Secondary structure	Helix	1.00	1.00	0.42	**0.03**
	Loop	1.00	1.00	0.42	0.08
	Sheet	1.00	0.55	0.69	0.71
Functional domain	Longitudinal	0.71	0.52	0.38	0.70
	Lateral	0.39	1.00	0.74	0.76
	MAP binding	0.17	1.00	**<0.01**	0.73
	GTP binding	0.63	0.39	0.61	1.00
	Lumen	0.31	0.34	1.00	0.61
	Intradimer	0.07	1.00	0.62	1.00
	Other	0.58	1.00	1.00	0.78

*p*-values listed for each test performed. Significant *p*-values are bolded.

**FIGURE 6:**
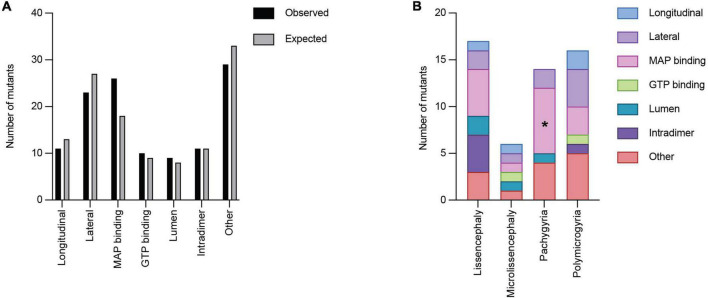
Quantifying *TUBA1A* mutations by functional region. **(A)** *TUBA1A* mutations were sorted by the functional domains (longitudinal, lateral, MAP binding, GTP binding, lumen, intradimer, or other) in which they reside. Observed and expected number of mutations were compared using a Chi-squared test [12.59 critical value when α = 0.05; X^2^ (df = 6, *N* = 119) = 5.177; *p* = 0.5]2). The expected number of mutations in each functional domain was calculated by determining the percentage of amino acids that reside in each domain, then multiplying that by 119 (the number of *TUBA1A* missense mutations known to date). This value represents the number of mutations that would be expected to appear in each domain if all 119 mutations were randomly distributed. **(B)** *TUBA1A* mutations were sorted by the primary cortical malformation resulting from each mutation (lissencephaly, microlissencephaly, pachygyria, or polymicrogyria). For each cortical malformation, the number of mutations in each functional domain was reported. A Fisher’s exact test was run for each cortical malformation category to determine if mutations were enriched in one functional domain over the others. Asterisk (*) on bar indicates *p*-value < 0.05 (pachygyria MAP binding *p* < 0.01).

We next asked whether *TUBA1A* mutations are grouped in specific regions of the three-dimensional tubulin structure that are known to play important roles in tubulin function. Based on structural and functional studies, we classified each mutant into the following ‘functional domains’: longitudinal, lateral, MAP binding, GTP binding, lumen, intradimer, and other. It is important to note that a mutation that is in the GTP binding region will also overlap with one of the other six functional domains. For simplicity and to ensure each category was mutually exclusive, if a mutation was located at a GTP binding residue, we classified it only as GTP. To test whether there is an overall enrichment of the 119 *TUBA1A* mutants in one of these functional domains we again used a Chi-squared test. We find there is no significant difference in the number of mutations found in each functional domain compared to what we would expect if the mutations were random [Figure 6A; 12.59 critical value when α = 0.05; X^2^ (df = 6, *N* = 119) = 5.177; *p* = 0.52]. Next, we asked whether within each malformation category there were significant differences in the number of mutations found in each functional domain. We used the 53 *TUBA1A* mutations with publicly available clinical analysis to perform a Fisher’s exact test. We find that the number of pachygyria mutations found in the MAP binding domain was significantly higher than would be expected if the mutations were randomly distributed (Figure 6B; Table 1; *p* < 0.01). This indicates that pachygyria mutations are more likely to be found in MAP binding domains compared to the other functional domains that we classified. It will be interesting to investigate further whether these mutations all impact the same MAP(s), and how disrupting these MAPs may result specifically in pachygyria. Mapping the mutations in this manner calls into question how we define functional domains. While we used structural and functional studies to define these regions, it is interesting to consider that other portions of the protein may have long-range effects that can impact these domains. Therefore, it is plausible that a mutation in one domain may also impact a domain on the far other side of the protein.

Importantly, these analyses suggest that we may reconsider how we view tubulinopathies. Microtubule regulation is a complex process that involves many moving parts that are connected across the tubulin heterodimer, and the role of a particular amino acid may not be limited to its local secondary structures or interacting regions of tubulin. In the next section, we synthesize our understanding of microtubule dynamics and the roles of specific regions of the tubulin heterodimer, and we argue that increasing our understanding basic tubulin biology will be important for understanding the field of tubulinopathies.

## Microtubule regulation

The association of MAP and tubulin mutations with cortical malformations in patients underscores the critical role of proper microtubule function during neurodevelopment. Therefore, studying the role of extrinsic MAPs and intrinsic tubulin dynamics, at the molecular level as well as in the process of neurodevelopment, is important for predicting how these individual mutations will disrupt neuronal cell biology and cortical formation at the tissue level. In this section, we will focus on recent advancements in the field’s understanding of tubulin intrinsic and extrinsic regulatory mechanisms. Additionally, we will argue how understanding these fundamental microtubule regulation studies will help us uncover molecular mechanisms and themes that persist across tubulinopathies.

### Tubulin conformations: From curved to straight and back

As discussed in the first section of this review, the series of conformational states adopted by the tubulin heterodimer are important aspects of both microtubule polymerization and depolymerization. The free, curved heterodimer in solution must add on to the microtubule plus-end (Buey et al., 2006; Rice Luke et al., 2008; Nawrotek et al., 2011), then subsequently straighten as it is assembled into the microtubule lattice (Nogales et al., 1999; Alushin et al., 2014) forming lateral bonds with neighboring protofilaments (Löwe et al., 2001). As these lateral bonds break apart during the process of depolymerization, tubulin heterodimers again splay out in a curved conformation and form the classically described “ram’s horns” (Mandelkow et al., 1991; Müller-Reichert et al., 1998). Understanding how the heterodimer shifts between these conformational states will thus be crucial in our understanding of intrinsic microtubule dynamics regulation.

The transition between these tubulin conformations states involves a network of structural rearrangements across the protein. Free α/β-tubulin heterodimer adopts an ∼12° bend at the intradimer interface, with a range between 9° and 18°, depending on the source of the tubulin structure (Campanacci et al., 2019). Recent work has identified various regions along the heterodimer that shift and/or contribute to these conformational states, with much of the work centered around specific β-tubulin rearrangements. Notably, there is a shift in helix 6 (H6) and H7, as well as the H6–H7 and T5 loops (Ravelli et al., 2004; Ayukawa et al., 2021). Additionally, a larger segment of β-tubulin containing the N-terminal domain and H11-H11’-H12 helices of the C-terminal domain also appear to shift with respect to one another (Ravelli et al., 2004). Further work has been done using mutations to disrupt β-tubulin H7 and has found that altering the structural regulation of this helix results in altered microtubule dynamics (Geyer et al., 2015; Ayukawa et al., 2021).

Additionally, a disease-causing mutant identified in the human β-tubulin isotype, *TUBB3*-D417H, results in a straightened heterodimer conformation and intrinsically faster microtubule polymerization rates *in vitro* (Ti et al., 2016). Similarly, disease-causing mutations identified in *TUBB4A* that result in dystonia and hypomyelination with atrophy of the basal ganglia, also impact tubulin conformation and microtubule dynamics (Krajka et al., 2022). However, these mutants had the opposite effect and instead promoted a curved tubulin conformation over a straightened state, with decreased microtubule polymerization rates *in vitro* and in cells (Krajka et al., 2022). These data highlight the significant contributions of individual regions of the heterodimer to conformation states, and how this can intrinsically alter microtubule dynamics.

In contrast to the progress made on the contributions of specific regions of β-tubulin to heterodimer conformation, decidedly less is known about α-tubulin’s influence. Our previous work indicates that a lissencephaly-causing mutation, *TUBA1A*-V409A, causes intrinsically faster microtubule polymerization rates despite decreased interaction with XMAP215/Stu2 TOG domains (Hoff et al., 2022). We propose a model in which the V409A mutation biases the heterodimer toward a straighter state that is more intrinsically favorable to microtubule polymerization and bypasses extrinsic regulation by TOG domains (Hoff et al., 2022) that recognize the curved tubulin state (Ayaz et al., 2012). This study likely indicates that there are regions of α-tubulin that are also crucial in establishing heterodimer conformations, however future in-depth studies will be required to identify and characterize these regions.

Particularly with the studies discussed regarding α-tubulin’s rule in dictating heterodimer conformation, it is clear that conformation states not only impact intrinsic microtubule dynamics, but also regulation conferred by extrinsic MAPs. In this next section we will discuss studies of MAPs that recognize and/or facilitate changes in tubulin conformation states, with a particular focus on how these two elements are significantly intertwined.

### Interactions between tubulin conformations and microtubule-associated proteins

In recent years, an increasing number of studies have identified MAPs that either preferentially bind to particular conformation states and/or control the transitions that occur between the curved and straight states of the heterodimer. One particular MAP of interest is XMAP215. As mentioned previously, XMAP215 is a microtubule polymerase (Brouhard et al., 2008; Al-Bassam et al., 2012) comprised of multiple TOG domains that preferentially bind curved tubulin (Ayaz et al., 2012; Ayaz et al., 2014). Additionally, XMAP215 proteins have a C-terminal basic region that is required for localization and tracking to the microtubule plus-end (Widlund et al., 2011; Byrnes and Slep, 2017). The prevailing model is that the TOG domains preferentially bind curved tubulin and stabilize an intermediate state for multiple rounds of addition at the microtubule plus-end. Therefore, while tubulin is able to polymerize on its own in an *in vitro* setting, microtubule polymerization rates are substantially increased when tubulin is in the presence of XMAP215 (Brouhard et al., 2008). Thus, recognition and stabilization of the free tubulin heterodimer by XMAP215 regulates its ability to assemble into microtubule polymer.

Interestingly, in the absence of free tubulin, the addition of XMAP215 also increases the rate of depolymerization (Brouhard et al., 2008). Similarly, the overexpression of Alp14, the XMAP215 homolog in fission yeast, spurs microtubule depolymerization (Al-Bassam et al., 2012). While the mechanism of how XMAP215 can also promote microtubule depolymerization is less understood, it is reasonable to assume that similar to other depolymerases discussed below, it involves recognition of a tubulin conformation state.

Op18/stathmin is another MAP family that has been shown to inducing and/or stabilize tubulin curvature (Gigant et al., 2000; Steinmetz et al., 2000). Stathmin binds to two individual tubulin heterodimers (Gigant et al., 2000; Steinmetz et al., 2000) and significantly increases microtubule catastrophe frequency (Belmont and Mitchison, 1996). As stathmin preferentially interacts with free tubulin heterodimers (Belmont and Mitchison, 1996), its pro-catastrophe mechanism is often described as sequestering free tubulin and preventing continued microtubule polymerization. While this is part of the mechanism, multiple works argue that the level of increase in microtubule catastrophe frequency observed in the presence of stathmin is also due to stathmin specifically targeting microtubule plus-ends to trigger catastrophes (Belmont and Mitchison, 1996; Steinmetz et al., 2000). These works highlight another MAP family that utilizes the curved conformation to recognize and induce changes in microtubule dynamics.

Beyond these MAPs that have been characterized to interact with particular heterodimer conformations, there are MAPs such as TOG-containing CLASPs and other kinesin polymerases that need to be further studied and may also recognize and/or influence tubulin conformations. However, it is clear from these studies that there is substantial interplay between tubulin conformations and MAP regulations, particularly that either factor can influence the other, or both can influence one another. Furthering our understanding of how these intrinsic and extrinsic modes of microtubule regulations work in unison will help us better identify how disruptions in these regulatory elements may ultimately lead to improper development and disease.

### Poisoning the network: How a subset of mutant tubulin can disrupt entire microtubule networks and cause tissue level defects

Almost all tubulinopathy mutations identified in patients to date appear as heterozygous, missense mutations (Hebebrand et al., 2019). A persistent question is how a subset of mutant tubulin can dominantly perturb microtubule networks in a way that ultimately disrupts a number of critical neurodevelopment events. Particularly for the mutations that appear subtle and do not significantly disrupt the hydrophobicity or charge of the amino acid at that residue, it is curious to consider how these mutants can act in such a dominant fashion.

In the few studies of individual tubulinopathy mutations, there has been an emphasis on mutants that would be predicted to disrupt MAP binding. For example, the *TUBA1A*-R402C/H tubulinopathy mutations are in a region predicted to be important for dynein binding (Mizuno et al., 2004). Accordingly, modeling these mutants in budding yeast α-tubulin specifically disrupts dynein, but not kinesin, function and in mice they are sufficient to dominantly disrupt neuronal migration (Aiken et al., 2019). However, MAP-binding may in some cases be accompanied by other consequences for tubulin function. The two β-tubulin mutations, *TUBB3*-D417H and -R262H, are located at residues near the kinesin binding site (Uchimura et al., 2006; Ti et al., 2016). While these mutant microtubules do decrease kinesin binding, they also bias the tubulin heterodimer toward a straightened conformation and accelerate microtubule polymerization (Ti et al., 2016). *TUBA1A*-V409I/A mutants disrupt binding of TOG domains, key components of the microtubule polymerase XMAP215 (Byrnes and Slep, 2017), but also accelerate microtubule polymerization (Hoff et al., 2022). These two latter cases further emphasize the point that the effects of mutations may be complex and impact both intrinsic tubulin activity and regulation by extrinsic factors. Therefore, understanding how intrinsic and extrinsic effects are linked will provide insight into how tubulinopathy mutations, even seemingly subtle substitutions, may have outsized effect on microtubule networks.

The studies of *TUBA1A*-V409I/A and *TUBB3*-D417H and -R262H also bring up a second important point—how do mutations in one region of the heterodimer impact structures or conformational changes that are not immediately adjacent to the affected residue? One consideration is that the normal tubulin activity relies on waves of conformational changes that are propagated across the heterodimer. Therefore, point mutations can have long-ranging, allosteric effects throughout the tubulin heterodimer itself, as well as along the entirety of the microtubule polymer. One example of this is threonine 238 of β-tubulin. A T238A mutation has been shown to form hyperstable microtubules, yet it is buried within the core of the tubulin protein (Thomas et al., 1985; Machin et al., 1995; Geyer et al., 2015). Despite residue T238 not being in direct contact with the E-site nucleotide, the T238A mutation prevents a conformational change that typically occurs in the lattice following GTP hydrolysis (Geyer et al., 2015). Thus an amino acid change far from the E-site creates microtubules that constitutively mimic a GTP-like state. Similarly, it is reasonable to consider that tubulinopathy mutations, such as *TUBA1A*-V409I/A and *TUBB3*-D417H/-R262H may have allosteric effects across the heterodimer that impact tubulin conformations and interactions with MAPs. Additionally, as these mutations make up less than half of the total α- or β-tubulin pool in the cell, it is also important to consider that these mutants may have long-ranging effects that impact neighboring heterodimers in the microtubule. More work will be needed to test this hypothesis. Testing the potential allosteric effects of individual disease mutants will likely require a combination of computational and structural studies, along with studies of tubulin activity *in vitro* and in cells that blend mutant and wild-type tubulin.

Interestingly, disease-associated mutations in other proteins have also been implicated in altering protein conformations. For example, the human protein ferritin, which stores iron inside cells, has at least eight unique disease-associated mutations that have been identified in patients (Cooper et al., 2005). These mutations have been linked to iron misregulation, cataract syndrome, basal ganglia disease, and a number of neurodegenerative diseases (reviewed in Levi and Rovida, 2015). These eight mutations are spread across the protein; however, it was found that each resided in regions of high rigidity and altered protein conformation dynamics (Kumar et al., 2015). On the other hand, mutations in more flexible regions that were not associated with disease were less likely to impact conformations (Kumar et al., 2015). Another example is a mutation in the prion protein that is associated with Creutzfeldt-Jakob disease, a neurodegenerative disease (Cooper et al., 2005). This mutation causes a conformational change in the C-terminal helix that inhibits the ability of the prion to form a functional dimer (Prabantu et al., 2021). Beyond these examples, an increasing number of studies are focusing on the allosteric impacts of single point mutations and how these ultimately lead to disease states (reviewed in Naganathan, 2019). We argue that there are lessons and tools to be learned from these areas that can be applied to tubulinopathy mutations to better understand the molecular mechanisms of these mutants.

## Concluding thoughts and future directions

Here, we review the link between tubulin structure, microtubules, and MAPs, and the roles they play in regulating microtubule dynamics. Importantly, we discuss how tubulinopathy mutations may disrupt multiple points of dynamics regulation. A major goal in the field of tubulinopathies is to identify mechanistic themes that can be used to accurately predict the impact of individual mutations at the molecular, cellular, and tissue levels. It is clear that the location and identity of tubulinopathy mutations is insufficient to explain disease phenotype. Therefore, we must recognize and better our understanding of the complexity and interconnectedness of microtubule regulators.

We propose a holistic approach to studying tubulinopathy mutations using improved computational models to predict allosteric impacts across the heterodimer and with MAP interactions. These approaches will be useful both in understanding mutations’ potential long-range effects, as well as for generating ideas about how specific regions of tubulin can impact activity from a distance. While computational simulations are an important first step, these ideas must then be validated using both *in vitro* reconstitution and experiments in cells that capture the complexity of isotypes and tubulin proteostasis. Together, using these tools in concert will be invaluable to advancing the field of tubulinopathies to ultimately identify common mechanistic origins of these neurodevelopment disorders so that we can better inform diagnoses and treatments.

## Author contributions

KH wrote the manuscript. AN helped write the manuscript. JM edited the manuscript and supervised the project. All authors contributed to the article and approved the submitted version.
